# Challenges of a GIS-based physical-geographical regionalization of Poland

**DOI:** 10.1007/s10661-023-11734-4

**Published:** 2023-08-31

**Authors:** Witold Piniarski

**Affiliations:** Landscape Ecology Research Unit, Adam Mickiewicz University, Poznań, Poland

**Keywords:** GIS, Landscape audit, Landscape contrast, Natural spatial units, Microregions, Regionalization of Poland

## Abstract

Poland’s traditional, i.e., non-GIS, regionalization needed to be updated for landscape audit purposes. Its spatial accuracy appeared insufficient, which led to the verification and adjustment of the existing physical-geographical mesoregions using GIS and high-resolution spatial data. In Poland, provincial landscape audits are part of implementing the European Landscape Convention to Polish law order, which led to the renewal of interest in the natural spatial division of the country. To date, there is no unified division of the entire country into microregions, which in Poland are commonly perceived as the most appropriate natural spatial units for local-scale landscape analysis and management. Microregions are lower-rank spatial units than already existing mesoregions. Both are distinguished by a homogenous landscape defined within the specific area by common physical-geographical characteristics of the land. Nevertheless, each is recognized at different scales and levels of generality. This paper focuses on reviewing the current challenges of the physical-geographical regionalization of Poland. Their fundamentals were identified through a systematic literature review. It also presents all the problems encountered within implementing GIS in the microregionalization procedure, which was already used for the Greater Poland Voivodeship landscape audit. In general, all traditional methodologies related to the physical-geographical regionalization of Poland require the introduction of GIS solutions to meet the current expectations from the country’s contemporary natural spatial division. The landscape contrast analysis method proved to be a promising method of GIS-based regionalization. It has the potential to become a universal solution to the existing problems with a unified physical-geographical microregionalization of Poland. However, some hard-to-overcome obstacles are related to the availability, collection, and processing of all required thematic spatial data. Nonetheless, it is highly expected to develop a universal procedure of microregionalization and distinguish low-rank units for the entire country.

## Introduction

Experiences with using physical-geographical regions in landscape management vary worldwide, and each country has specific landscape protection instruments developed over many decades (Majchrowska, 2018). The European Landscape Convention (ETS No. 176) sets great stores by identifying and assessing landscapes. Additionally, it is the first international treaty that envisages all aspects when planning European landscapes, significantly impacting current research trends and governmental strategies within EU countries (Pătru-Stupariu & Nita, 2022). In addition, the treaty aims to promote the protection, management, and planning of European landscapes of individual countries and organize cooperation on widely understood landscape issues (Déjeant-Pons, 2006; Olwig, 2007). Challenges of implementing participation in the European Landscapes Convention resulted in unique approaches to environmental management among signatory countries (Jones & Stenseke, 2011; Scott, 2011; Roe, 2013; De Montis, 2014; Dempsey & Wilbrand, 2017; Dovlén, 2016; Trykacz & Bernat, 2022; Civitarese Matteucci & Cartei, 2022).

In Poland, studies connected with the landscape audit made researchers all over the country work on the guidelines of the process, which recently seems to be an overwhelming direction of Polish landscape ecology applications (Degórski et al., 2014; Solon et al., 2014, 2015; Myga-Piątek et al., 2015; Kistowski et al., 2018; Kistowski, 2019; Chmielewski, 2020; Piniarski, 2020; Macias & Bródka, 2021). A renewal of the interest in the physical-geographical regionalization of Poland arose with the introduction of the Act of 24 April 2015, amending certain acts in relation to strengthening tools of landscape protection instruments (Journal of Laws of the Republic of Poland, 2015, item 774), which resulted from the implementation of the European Landscape Convention and first included landscape audit regulations in the Polish legal order. Adaptation of a coherent EU landscape policy is also visible within strategic documents, e.g., the National Spatial Management Concept 2030 (NSDC 2030, 2012), which includes “Shaping spatial structures which contribute to achieving and maintaining the high quality of Poland’s natural environment and landscape.” For Poland, following the recommendations in the proposal of methodology for preparing landscape audits (Pukowiec-Kurda & Myga-Piątek, 2017; Solon et al., 2014), base materials for delimitation and identification of the landscapes include physical-geographical mesoregions and, in the future, should also include microregions, which have not yet been developed for the whole country area.

Previous commonly accepted physical-geographical division of Poland by Kondracki and Richling (1994) has proven insufficient to work with detailed, high-resolution data, e.g., in determining priority landscapes as one of the primary goals of the landscape audit. As a result, its newest revision was developed by Solon et al. (2018), mostly maintaining regional boundaries from the original version (Kondracki & Richling, 1994). Generally, it was focused on minor adjustments and improving the course of the existing boundaries (Solon et al., 2018; Richling et al., 2021). Although the spatial accuracy of the physical-geographical division of Poland has radically improved at the time, it still includes only relatively large spatial units. To date, there is no unified physical-geographical division into spatial units in a rank lower than mesoregions. Therefore, it is still highly expected to distinguish the complementary subdivision of the entire country into spatial units in a rank of microregions, which have been clearly defined as the most appropriate basic analytical fields for a high-precision landscape analysis at the local scale (Balon & Krąż, 2013). The paper presents difficulties in distinguishing physical-geographical microregions in Poland, in the example of the area of the Greater Poland Voivodeship. The procedure implies processing large spatial datasets, including tens or hundreds of millions of data objects. The most time-consuming single operation took up to 56 h of uninterrupted computer processing, while dozens of steps were required to complete the microregionalization. The concept scheme of the developed procedure consists of a few essential stages that include numerous geometrical operations and spatial data analyses, which require various GIS processing tools. Therefore, much effort in the following paper was devoted to methodological problems, the data used, and its processing within the developed GIS-based microregionalization procedure.

## Materials and methods

### Theoretical background

The basic concept and methodological solutions adopted in this work follow the major guidelines proposed in the pre-GIS era, i.e., from the mid-1940s to mid-1970s, at first by J. Kondracki and later by A. Richling (Kondracki, 1946a, b, 1955, 1961, 1965, 1968, 1969, 1976; Richling, 1976). Even though the basics of physical-geographical regionalization were established in the first 30 years from the initial Kondracki’s works, the author had not perceived his work as finished. Therefore, the regionalization was first further developed by J. Kondracki himself (1977, 1978, 1988, 1994) and afterward in collaborations with J. Ostrowski and A. Richling (Kondracki & Ostrowski, 1968, 1973–1978, 1994; Kondracki & Richling, 1983, 1994). In later elaborations, Kondracki (1998, 2000, 2002) stated that the regionalization of the country had been completed. The map from the Atlas of the Republic of Poland (Kondracki & Richling, 1994) became the new standard for the following decades, i.e., until the development of its latest GIS revision by Solon et al. (2018).

**Fig. 1:**
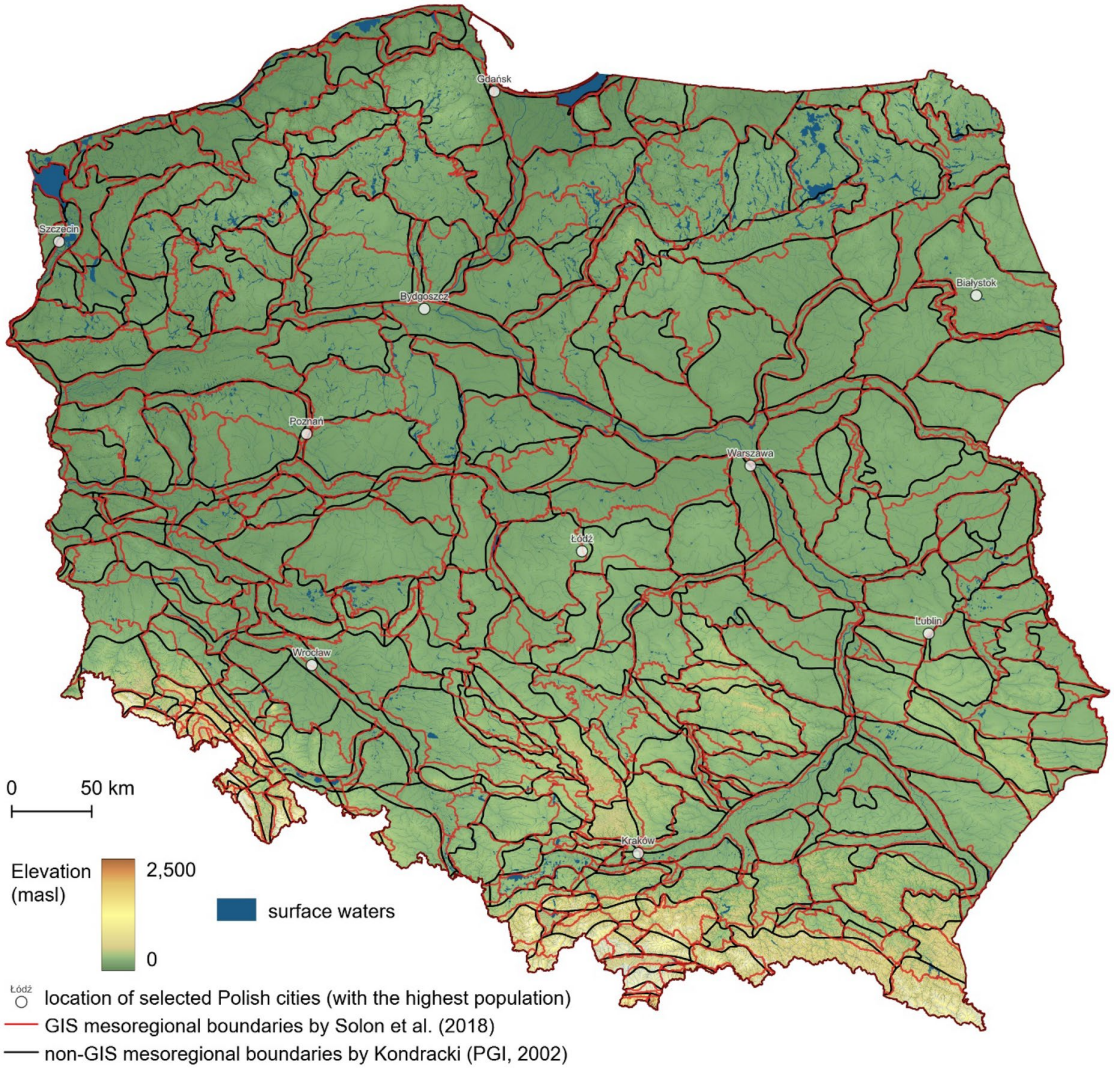
Comparison of the former (non-GIS) and the contemporary (GIS) version of mesoregional boundaries of the physical-geographical regionalization of Poland

The use of GIS tools and high-resolution spatial data has been one of the frequently repeated topics of recent studies connected with the physical-geographical regionalization of Poland (Kistowski et al., 2018). In the pre-GIS era, manual mapping techniques substantially limited regionalization, resulting from no access to digital data and GIS processing tools, using only analog cartographic materials and hand drawing (Richling, 2014). In this connection, analog tools and manual delineation methods were causing some inevitable approximations and inaccuracies in the course of the original, non-GIS regional boundaries. The spatial accuracy of the pre-GIS physical-geographical division of Poland by Kondracki and Richling (1994), which was developed in the overview map scale, i.e., 1:200,000 or lower, turned out to be insufficient for the program of identification cataloging and evaluation of Polish landscapes to implement the European Landscape Convention (Council of Europe, 2000) into the Polish legal order. This situation has forced the necessity of verification and adjustment of the existing boundaries with GIS, which led to radical spatial accuracy improvements of the previous regionalization by Kondracki and Richling (1994), i.e., joint verification and adjustment of the physical-geographical division of Poland to the scale of 1:50,000 by Solon et al. (2018). Detailed adjustments of the boundaries were performed with GIS tools based on a high-resolution digital elevation model (DEM) and geological and geomorphological datasets (Solon et al., 2018). The main idea was to use up-to-date GIS tools and high-resolution digital data while respecting all the earlier developed theoretical assumptions by J. Kondracki and A. Richling, i.e., to maintain the basic concept of the previous version of the regionalization. A comparison of the digitized, non-GIS physical-geographical division of Poland by Kondracki from the Central Geological Database (CBDG) (PGI, 2002) with its latest GIS revision by Solon et al. (2018) shows many similarities to the original course of the mesoregional boundaries (from 1994) but also indicates a wide range of introduced spatial accuracy improvements (Fig. 1).

Geographic information systems (GIS), high-resolution spatial data, and digital computing capabilities have allowed researchers to significantly increase coverage and enhance their elaborations’ spatial accuracy and precision. The border analysis method was initially developed and introduced to Polish studies related to physical-geographical regionalization by A. Richling (1976). One of the early GIS incarnations of the border analysis method brought the concept of the landscape contrast map as a combination of many overlapping spatial data layers containing typological characteristics of selected physical-geographical components of the environment (Kozieł, 2006). The border analysis method was further developed with the addition of more compounded GIS analyses and evolved into the overlapping border method, which can be a baseline to delimitate low-rank natural spatial units, i.e., physical-geographical microregions, using landscape contrast maps (Kistowski & Szydłowski, 2014, 2015; Pietrzak, 2010, 2020).

In constructing a landscape contrast map, the extensive study area and complex geometry of all used spatial data make manual delineation of precise linear borders out of this irregular, seemingly chaotic pattern problematic with the conventional approach to landscape contrast analysis. Traditionally, in the pre-GIS era, determining boundaries strictly from a graphical analysis of analog cartographic materials, i.e., maps printed on a specific scale, led to some inevitable simplifications and approximations of their linear course (Richling, 2014). They concern the subjective perception of the analyst, limitations of manual cartography techniques, and usage of analog spatial data, i.e., printed maps. However, the expert knowledge and experience of the analyst cannot be underrated and should also be considered in this matter. An excellent example is Poland’s original non-GIS physical-geographical regionalization (Kondracki & Richling, 1994). First, it gained universal acceptance by the government, researchers, and many practitioners (Kistowski et al., 2018). Second, despite some far-reaching changes in the detailed course of the regional boundaries, in most cases, the previous system of natural spatial units was preserved in the later-developed GIS version (Solon et al., 2018). All of this indicates the high substantive value of the original 1994 regionalization of Poland, which undoubtedly was one of the most outstanding achievements of Polish physical geography of the pre-GIS era.

The main assumptions of Poland’s contemporary physical-geographical division were established when the physical geography and landscape studies of Central and Eastern Europe showed the trend of the so-called contrastivity principle. At the time, studies of integral physical-geographical landscapes were primarily focused on the homogeneity of mapped lands and the aspect of landscape contrastivity along contact zones of contrasting, contiguous landscapes (Mil’kov, 1979). It is still a vital assumption to physical-geographical regionalization, which is related to the concept of ecotones and physiographic, landscape-ecological approaches to landscape divisions, where landscape boundaries are considered a kind of spatial zone with smooth, gradient changes in specific characteristics of the environment (Chmielewski & Kułak, 2016; Chmielewski et al., 2019). The so-called idea of marginal zones (Neef, Ed., 1978; Pietrzak, 2010, 2020), which occur at the junction of considered landscape characteristics, i.e., delimitation criteria, is crucial to the landscape contrast analysis method and delineation of natural spatial boundaries (Piniarski, 2020, Macias & Bródka, 2021). Within each marginal zone, the increasing number of contacts of boundaries of the distinct homogeneous areas, from the view of each selected delimitation criterion, indicates higher landscape contrast and sharpness of the border at the specific segments of it (Przewoźniak, 1987; Richling, 1992; Solon & Myga-Piątek, 2018).

It is necessary to distinguish between linear, i.e., crisp, sharp artificial boundaries of administrative units, and indeterminate, i.e., fuzzy, vague boundaries of natural spatial units (Vogt et al., 2012). Natural boundaries are based on the physical-geographical features of the land, while artificial boundaries are set arbitrarily by national governments or in border treaties between countries (Caflisch, 2010). The problem is that the boundaries of natural spatial units largely do not coincide with administrative units, except for some examples of riverbanks, mountain peaks, or other natural barriers that have visibly influenced the course of artificial borders (Fall, 2010). Linear boundaries take the form of lines regardless of the spatial scale in which they are examined, while zonal boundaries depend on this scale and can take the form of lines or stripes of various widths. In general, almost all artificial boundaries, i.e., country borders (excluding demarcation zones) or administrative borders, are linear, in opposition to natural boundaries, which can be marked as lines only with a specific adaptation of delineation method and with certain cartographic generalization (Bański, 2010). Since all natural boundaries depend on the assumptions of their delineation, they should not be considered strictly as lines but rather as kinds of spatial zones, stripes, or buffers. They can be perceived as linear objects only at a specific spatial scale. All this contradicts the needs of Polish local governments regarding the strict and precise delineation of natural boundaries for local-scale environmental management, e.g., conducting landscape audits and determining priority landscapes.

### Study area

According to the so-called Landscape Act, i.e., the Act of 24 April 2015 on amending certain acts in relation to strengthening landscape protection instruments (Journal of Laws of the Republic of Poland, 2015, item 774), each voivodeship (province) is obliged to conduct the audit on their territory at least once every 20 years, which aims to help the Polish legislator provide more effective landscape protection tools (Karpus, 2016; Krajewski & Solecka, 2019; Szlachetko, 2019). It is significant that the document entitled “Identification and assessment of Polish landscapes – stages, and methods of actions within the landscape audit in the administrative regions” (Solon et al., 2015) directly points out the physical-geographical units in the rank of microregions as the most appropriate test fields for the landscape audit. All this makes the administrative units in the rank of provinces one of the most obvious choices for a microregionalization area in Poland, especially when the focus is also on the practical aspects of the study and the possibility of using its results by the local self-government authorities for sustainable environmental management within their territory.

**Fig. 2:**
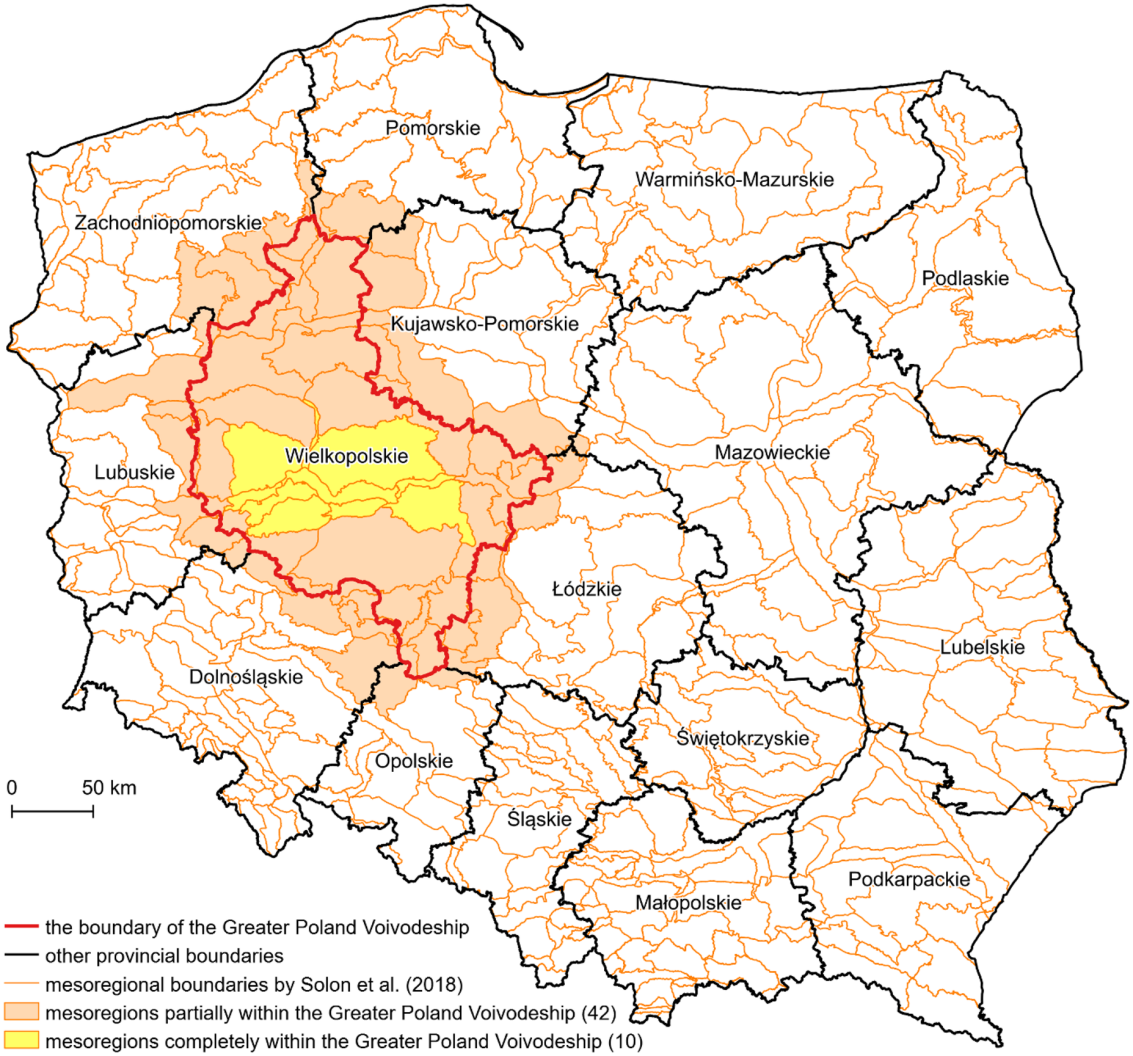
Location of the study area on the background of the physical-geographical mesoregions of Poland, showing the spatial continuity of natural spatial units outside the administrative boundaries of provinces

Greater Poland is a historical region of west-central Poland. Currently, it lies mainly within the borders of the Greater Poland Voivodeship, also known as Wielkopolskie, which is the second largest of all 16 provinces (voivodeships). It covers an area of almost 30 thousand sq. km, i.e., close to 10% of the country’s territory. According to the valid physical-geographical division of Poland (Solon et al., 2018), within the limits of the Greater Poland Voivodeship are 42 physical-geographical mesoregions. However, only 10 of them lie entirely within its boundaries. The study area is a perfect example of a dilemma in distinguishing the natural and artificial borders of some territories (Fig. 2). This connects with a spatial continuity of natural spatial units, e.g., physical-geographical regions and other nonartificial territorial identities, i.e., historical, cultural, or economical, outside administrative units, i.e., communities, counties, provinces, and countries (Melnychuk & Gnatiuk, 2018).

## Spatial data

### Thematic resources

Public access to current geodetic and cartographic information in Poland is provided mainly by the Head Office of Geodesy and Cartography (GUGiK), which is also responsible for creating modern spatial data infrastructure, i.e., geoportal data and services, following the guidance of the EU INSPIRE Directive of 2007 (Directive 2007/2/EC). In addition to the Main Center for Geodesic and Cartographic Documentation (CODGiK), which tasks and resources were taken over by GUGiK in 2017, there are still provincial (WODGiK) and county (PODGiK) geodesic and cartographic centers. National geodetic and cartographic data are provided online and free of charge at the National Geoportal (https://geoportal.gov.pl), e.g., as a part of the State Geodetic and Cartographic Resource (PZGiK).

In general, Polish thematic map resources are well-developed and consist of much spatial data focused on various components of the environment. However, their spatial coverage is commonly limited to selected administrative units or map sheets by the index of Polish topographic maps rather than natural spatial units. Moreover, they are often available with various spatial accuracies and precisions and distributed in diverse data types and formats. As has already been stated, the spatial accuracy and precision of physical-geographical boundaries substantially depend on the source material, i.e., selected criteria and corresponding spatial data. Their quality can be determined by multiple cartographic characteristics, i.e., theme content, spatial coverage, spatial accuracy, and data type and distribution format (Piniarski, 2020). All the available thematic spatial data are the results of many years or even decades of work by many national institutions and research development centers, e.g., the Polish Geological Institute (PGI) and Institute of Soil Science and Plant Cultivation (IUNG), together with many other science-related institutions, i.e., Polish Academy of Sciences (PAS) and Institute of Meteorology and Water Management (IMGW), as well as many Polish universities and other related academic institutions. These are the most reliable cartographic materials available for each component of the environment, resulting from comprehensive field studies and many expert opinions, elaborated with multilevel verification and validation by the broadly understood scientific community, decision-makers, and practitioners.

### Spatial data and regionalization criteria

Adequate spatial datasets were selected by reviewing the thematic map resources available within the study area, according to previously described theoretical and formal-legal aspects of physical-geographical regionalization in Poland. In addition, to ensure relatively high constancy and immutability of the distinguished natural spatial units, selected delimitation criteria refer to the concept of environmental stability (Moss, 1999; Solon, 1994), which indicates the more persistent abiotic components of the environment, such as landforms, geological structures, and climatic regimes, as more readily defined and easier to conceptualize. The spatial datasets used include surface geological characteristics, genetic interpretation of landforms, and primary topographic attributes, i.e., relief types and terrain slopes (Kot, 2014; Nita, 2010; Urbański, 2011). Other considered environmental characteristics were related to waters, soils, and potential natural vegetation (PNV), representing the study area’s local climate characteristics by determining the most adapted plant species for a definite ecotope (Matuszkiewicz, 2008).

The data layer of subsurface geological structures was based on the Detailed Geological Map of Poland at the scale of 1:50,000 (SMGP), primarily developed by PGI in 1956–2009 as a serial map divided into 1069 sheets covering the entire territory of Poland. All SMGP sheets are available online within the CBDG GIS resources (https://gis.pgi.gov.pl) as a Web Map Service (WMS); moreover, within the PGI structures exists the National Geological Archives (NGA), which surveys, collects, maintains, and makes all PGI data available also stationary (at Warsaw headquarter and regional branches across Poland). Each SMGP sheet includes detailed documentation, comprehensively explaining the geological structure and considering lithology, genesis, and stratigraphy of formations, as well as geomorphology and tectonics. Out of 130 sheets within the study area’s extent, only 100 were initially available as vector data. The remaining 30 were acquired in a raster data format as digital scans of previously published printed maps.

**Fig. 3:**
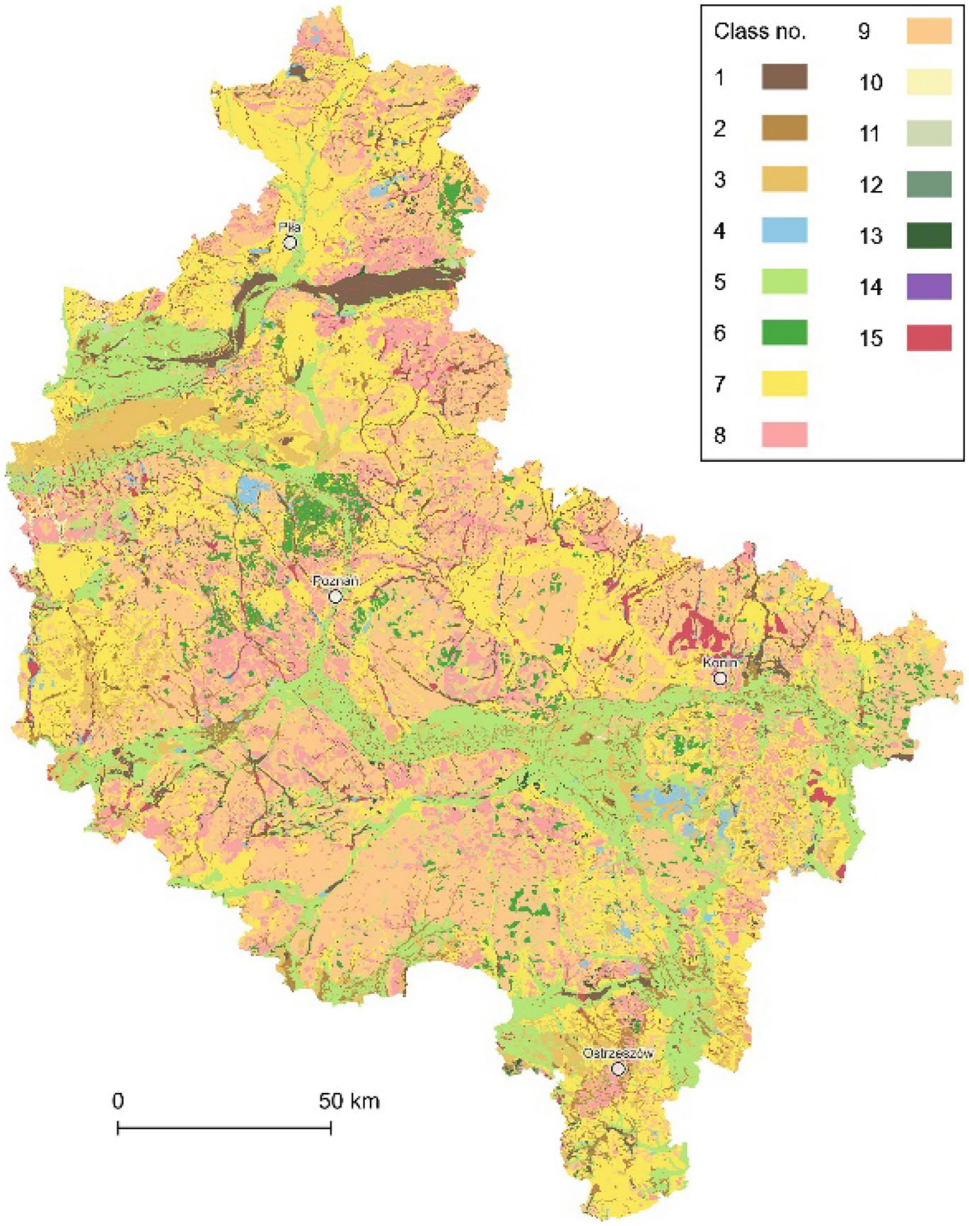
The data layer of subsurface geological structures (15 classes)

First, all the complimentary rasters were vectorized. Second, all the SMGP’s sheets were unified as vector data and merged into a single spatial data layer with 15 classes of subsurface geological structures (Fig. 3), according to the simplified legend of the SMGP, i.e., 1, peats and other organic sediments; 2, alluvial, slopewash and valley bottom deposits; 3, eolian accumulation sands and loess-like deposits; 4, lake sediments; 5, river sediments; 6, eluvial deposits; 7, fluvioglacial sands and gravels; 8, sands, gravels, and boulders of glacial accumulation; 9, boulder clays of glacial accumulation; 10, glacial tills; 11, subglacial deposits; 12, alluvial sediments; 13, tertiary sands, gravels, clays, and brown coal deposits; 14, Mesozoic deposits; and 15, no data and mixed geological materials (most frequent within heavily transformed areas, e.g., near the Konin Coal Mine).

**Fig. 4:**
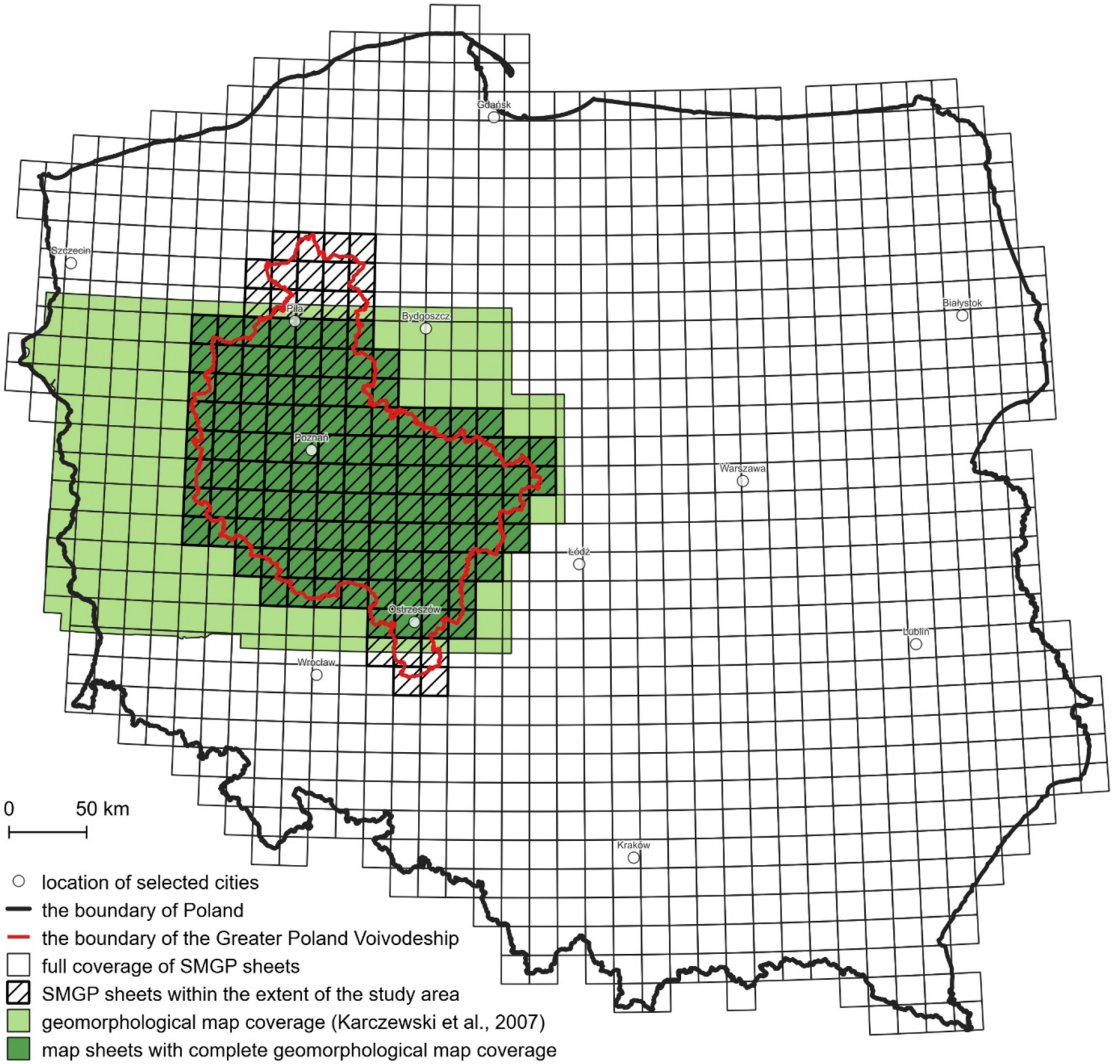
Spatial coverage of the SMGP sheets and the geomorphological map within the Greater Poland Voivodeship

Geomorphological drafts at the scale of 1:100,000 are attached to each SMGP sheet as part of the mentioned additional cartographic documentation. The spatial coverage of the used geomorphological map of the Wielkopolska-Kujawy Lowland (Karczewski et al., 2007), which was the basis for the development of the data layer of the genetic interpretation of landforms, is limited to the extent of 113 out of 130 SMGP sheets in the central part of the study area. Therefore, the geomorphological drafts were used to complete the data layer within the missing northern and southern parts of the study area, i.e., 13 sheets north of the city of Piła and 5 south of the town of Ostrzeszów (Fig. 4).

**Fig. 5:**
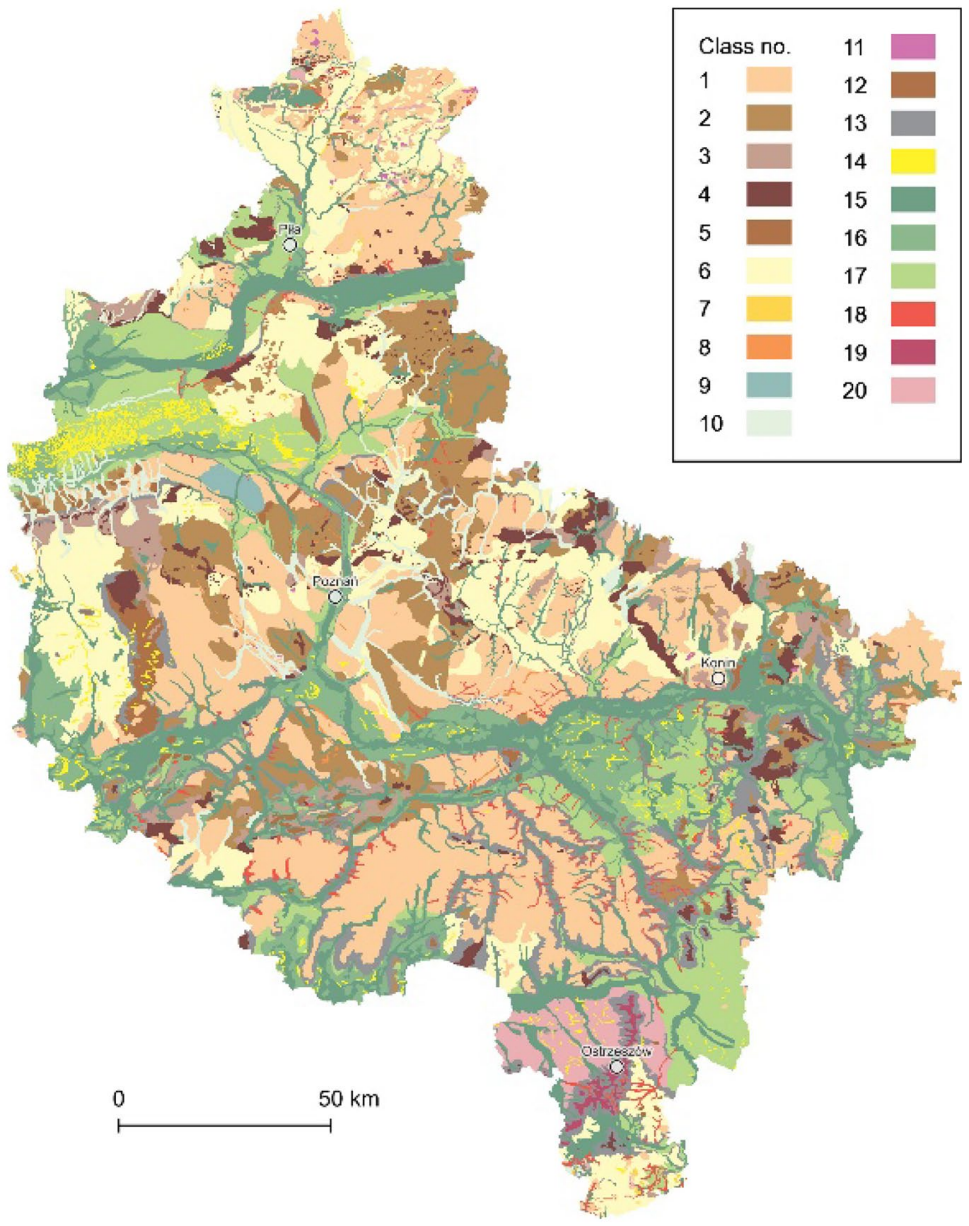
The data layer of the genetic interpretation of landforms (20 classes)

The resulting data layer consists of 10 geomorphological landform classes (Fig. 5), i.e., 1, flat moraine uplands; 2, wavy moraine uplands; 3, hilly moraine uplands (hummocky moraines); 4, end-morainic hillocks; 5, moraine embankments; 6, outwash sand plains (sandurs); 7, kames; 8, eskers; 9, marginal plains; 10, glacial tunnel valleys; 11, dead-ice features; 12, monadnock hills; 13, long slopes; 14, eolian landforms (dunes); 15, floodplains and low terraces; 16, middle terraces; 17, high terraces; 18, valleys and ravines cutting the upland; 19, flat-topped hills/mountains of the elder glaciations; and 20, periglacial plains.

**Fig. 6:**
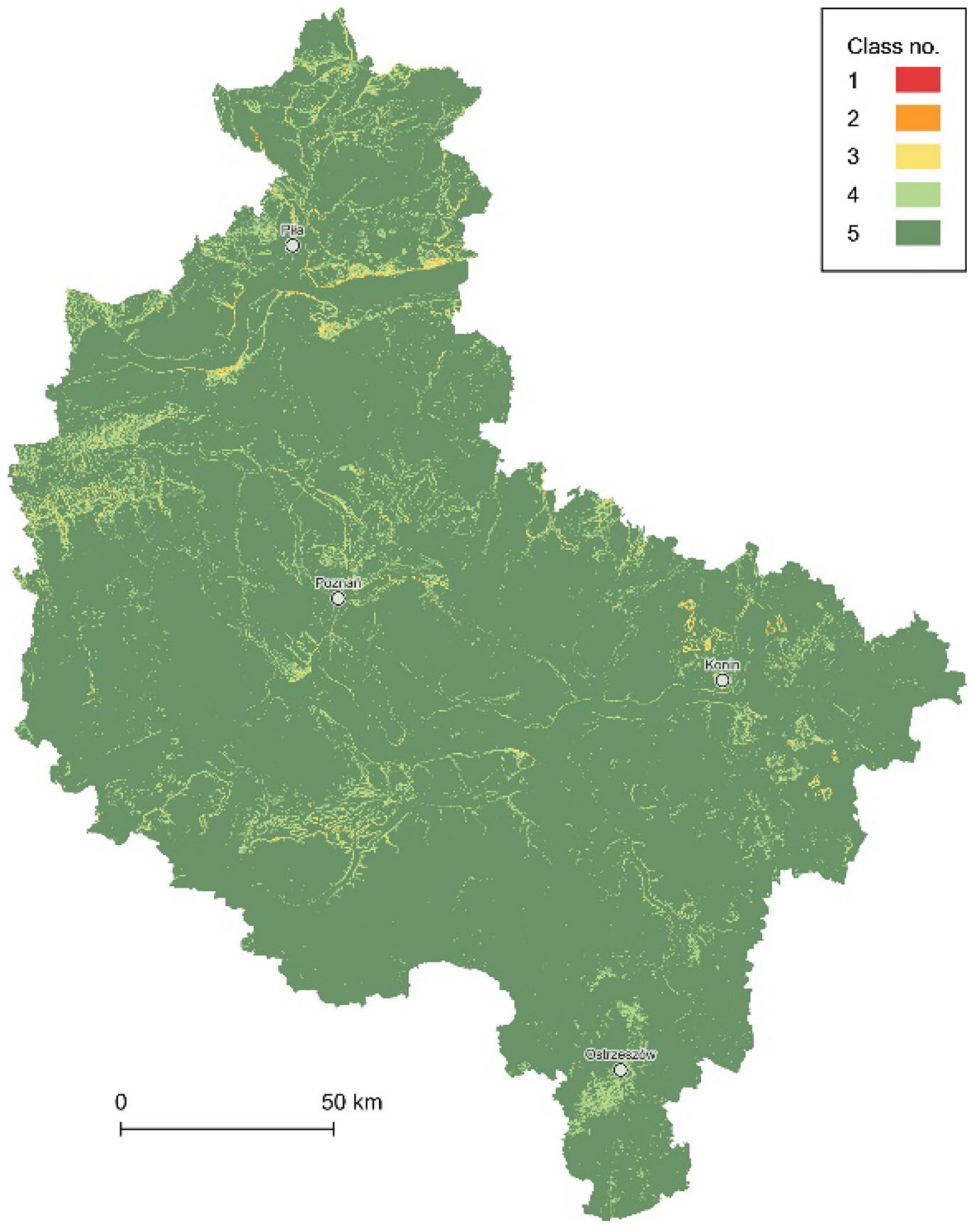
The data layer of the terrain slopes (5 classes)

The terrain slope data layer was developed with the source digital terrain model (DTM), available within the National Geoportal at a 1-m resolution for all the territory of Poland. The source DTM was based on light detection and ranging (LiDAR) measurements obtained from airborne laser scanning (ALS) by GUGiK. Initially, it consisted of 6130 ASCII XYZ files in text data format (TXT), containing over 873 GB of data, which made their processing computationally demanding. Simultaneously, the DTM resolution of 1 m was redundant compared to the resolutions of the other data used within the research, considering all the differences, i.e., their varied cartographic characteristics. As a result, the DTM was simplified to a 50-m grid using Python programming tools, and all further analyses proceeded in the net of square test fields of the same grid size (Piniarski, 2020). Terrain slope classes (Fig. 6) were developed by morphometric analysis of the DEM, following 5 percentage classes defined earlier by Richling (1979), i.e., 1, more than 20; 2, 10.01–20.00; 3, 5.01–10.00; 4, 2.01–5.00; and 5, less than or equal to 2.00 (%).

**Fig. 7:**
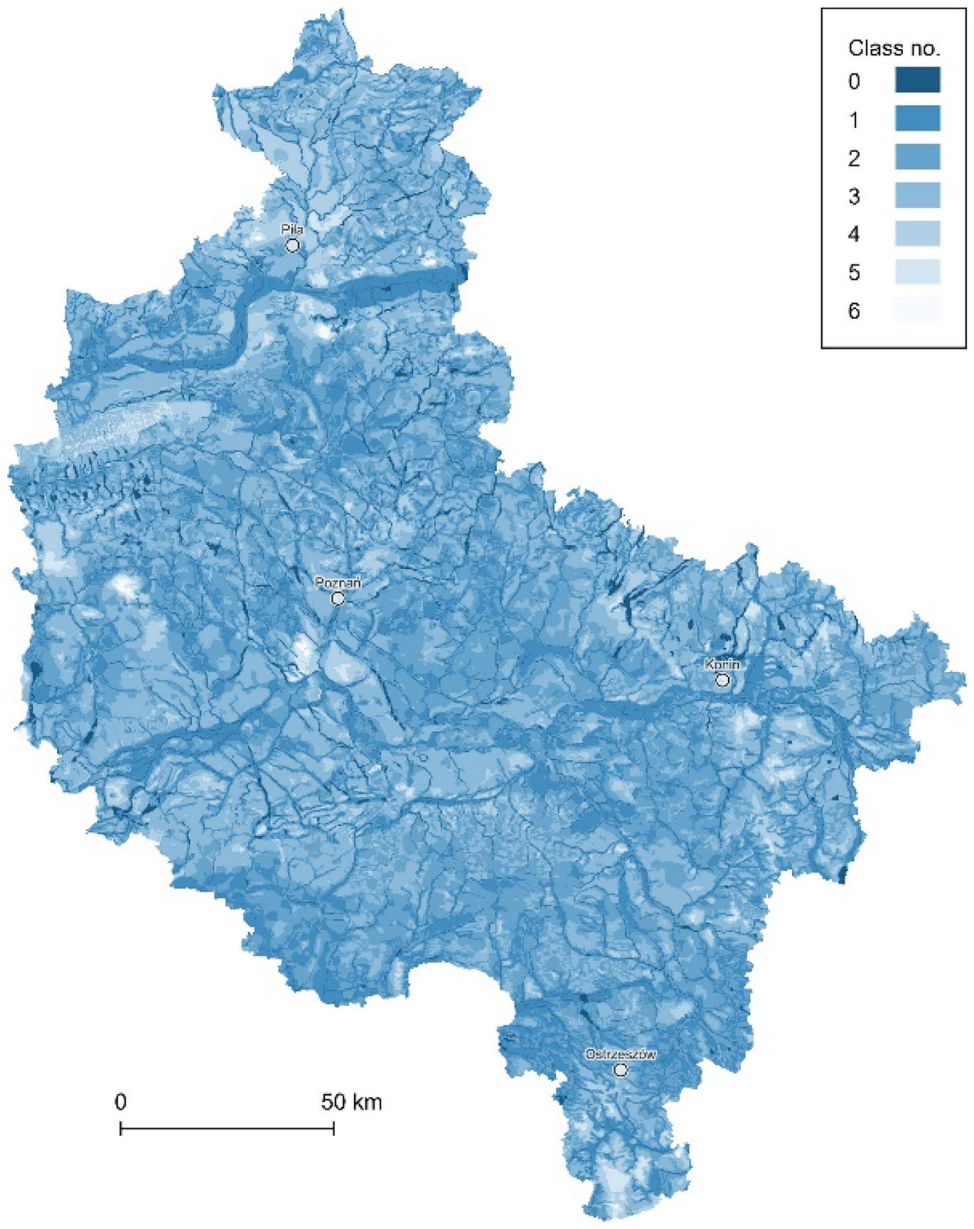
The data layer of the surface waters (class no. 0) and depth to groundwater (classes no. 1–6)

The surface water data used, one of the land cover object classes within the BDOT10k (part of the PZGiK), present bodies of all the stagnant and flowing waters within the study area with the accuracy corresponding to the topographic map at the scale of 1:10,000. The depth to groundwater was calculated by interpolating isolines of groundwater level from the numeric version of the hydrographic map of Poland at the scale 1:50,000, whose various versions are also available as WMS within the National Geoportal. The developed waters data layer presents all surface water bodies (class no. 0) and 6 classes of groundwater level depth (Fig. 7), with values specified in meters below ground level (mbgl), i.e., 1, less than or equal to 1.00; 2, 1.01–2.00; 3, 2.01–5.00; 4, 5.01–10.00; 5, 10.01–20.00; and 6, more than 20. Selected classes correspond to the depth ranges distinguished within the GIS database of the Hydrogeological Map of Poland at a scale of 1:50,000 (Fert et al., 2011).

**Fig. 8:**
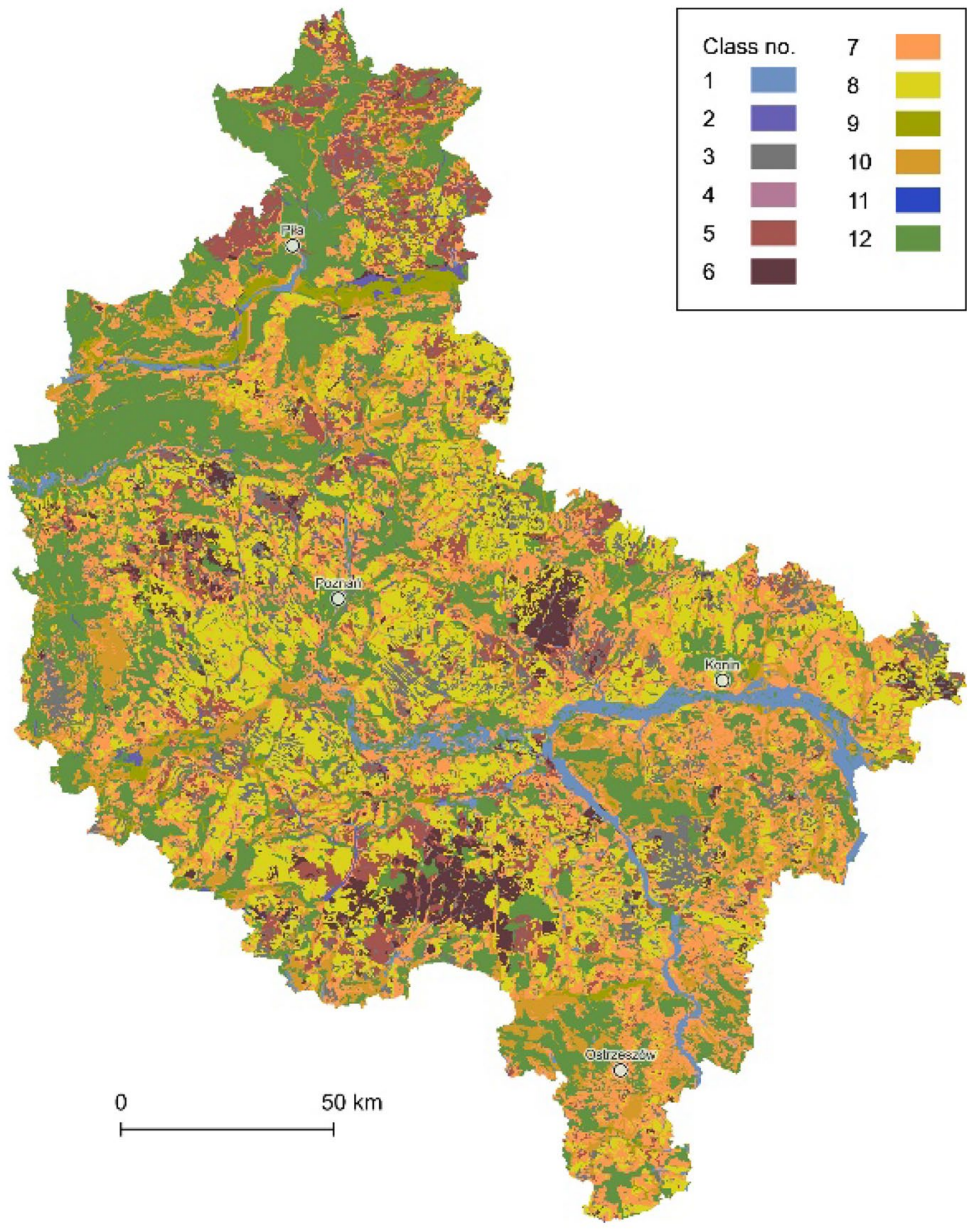
The data layer of the genetic soil types (12 classes)

The data layer of genetic soil types was built on the numeric soil and agricultural map at the scale of 1:100,000, which was acquired on request directly from IUNG and is not distributed online. However, it is worth noting that for the selected parts of Polish provinces, the data are also available at the scale of 1:25,000, or even 1:5,000, and distributed as part of the cartographic materials of each local WODGiK’s geodetic and cartographic resources. Nevertheless, in the case of the Greater Poland Voivodeship, more accurate vector-based data are not available within the entire study area. Eventually, the genetic soil types data layer consisted of 14,189 data objects grouped within 12 classes (Fig. 8), i.e., 1, typical ordinary alluvial soils; 2, muddy soils; 3, typical black earths; 4, ordinary rendzinas and pararendzinas; 5, leached brown soils; 6, ordinary brown soils; 7, different types of sandy soils (developed from glaciofluvial, eolian, and older alluvial sands); 8, clay-illuvial soils; 9, peat soils; 10, murshic soils; 11, degraded black earths; and 12, soils under forest and other unspecified types. The presented classification and all specified soil types correspond with the Polish Soil Classification (Kabała et al., 2019).

**Fig. 9:**
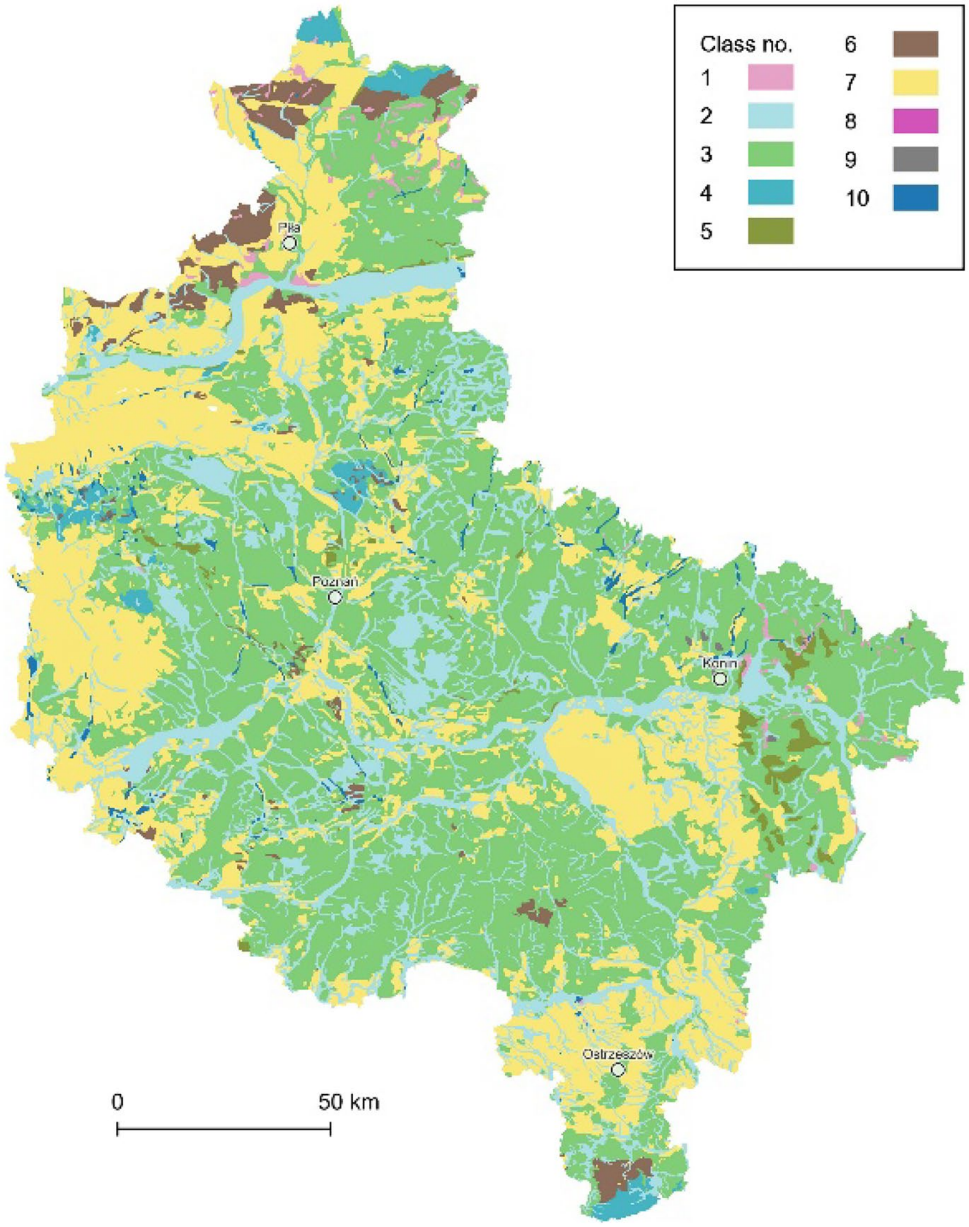
The data layer of the potential natural vegetation communities (10 classes)

Community groups of the potential natural vegetation (PNV) were derived from the map at a scale of 1:300,000 (Matuszkiewicz, 2008), for which 16 raster sheets can be downloaded from the Institute of Geography and Spatial Organization (IGSO) PAS website (https://www.igipz.pan.pl/potential-vegetation-dge.html). However, on direct request, the author provided the vector PNV data for the microregionalization. Within the study area, second-class communities groups count 10 classes, including a separate surface waters class (Fig. 9), all of them with a total of 2884 data objects, i.e., 1, lowland alder and birch swamp or peat forests; 2, deciduous alluvial forests, as well as hygrophilous broad-leaved and forb-rich forests on the groundwater soils; 3, lowland mesophilous broad-leaved forests, mainly with oak and hornbeam predominant; 4, lowland beech forests; 5, thermophilous oak forests; 6, acidophilous oak and beech-oak mixed forests; 7, oligotrophic acidophilous pine forests; 8, mossy bog vegetation with dwarf shrubs; 9, devasted environment vegetation, succession unknown and areas without vegetation; and 10, surface waters.

## Regionalization procedure

With the use of GIS, it is possible to construct a landscape contrast map and precisely quantify the importance of the border at the specific segment of it (Kistowski & Szydłowski, 2014). On the map, the greater the number of overlapping boundaries, the higher the landscape contrast along the specific border segment. In general, with higher values of the landscape contrast comes greater landscape variety and more variability between two opposite sides of the border, i.e., higher sharpness of the specific border segment. Lower values of the landscape contrast mean less variety and more similarities between two opposite sides of the border, i.e., higher fuzziness of the specific border segment. Without a GIS-supported analysis, clearly defining their sharpness would not be possible. Therefore, it is crucial to draw the linear course of the boundaries at the segments with the highest landscape contrast and the intended spatial accuracy.

**Fig. 10:**
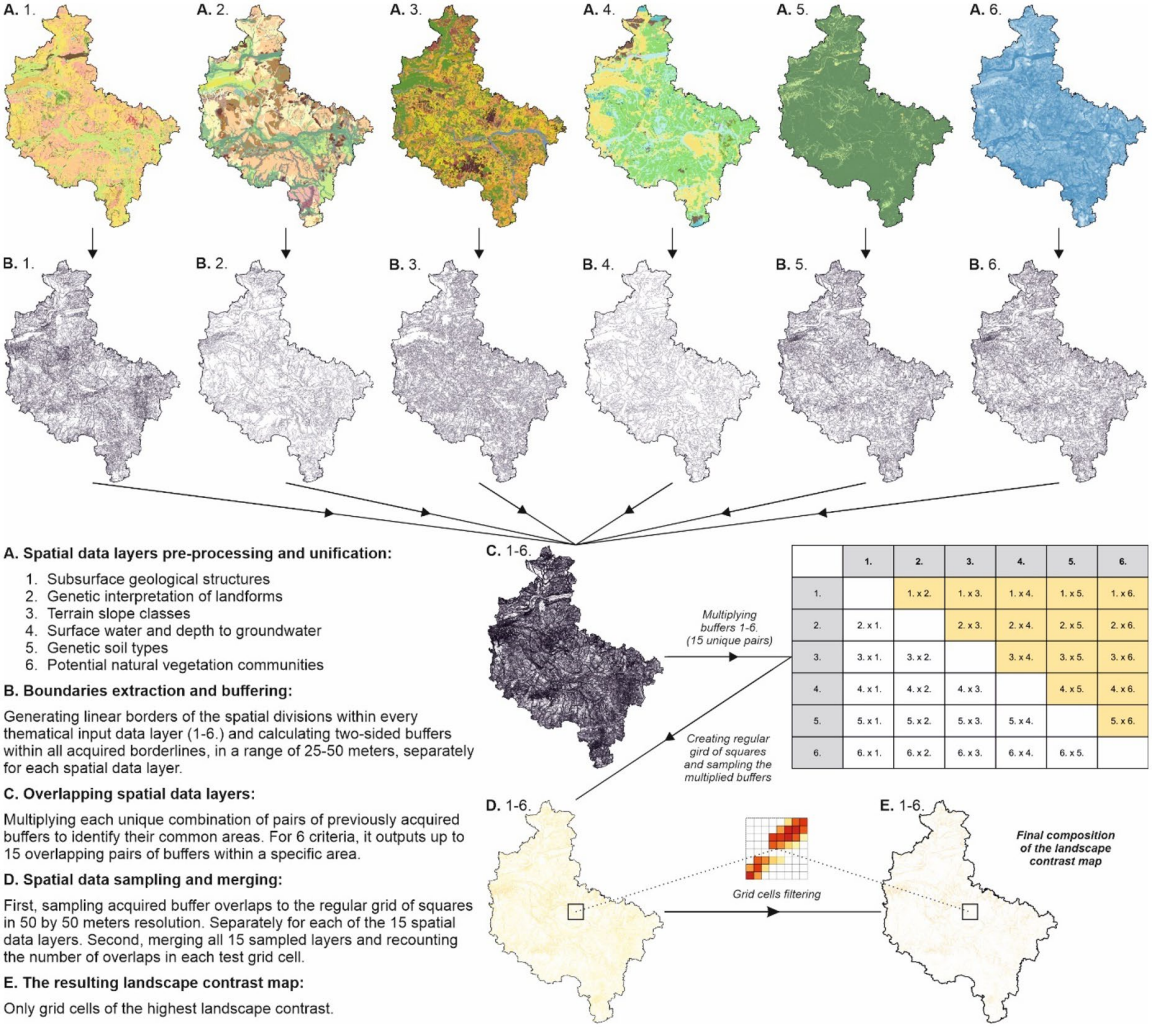
Stages of constructing the landscape contrast map of the Greater Poland Voivodeship

First, in QGIS, all the gathered spatial data were preprocessed, i.e., merged and unified as shapefile (SHP) vector data layers for each regionalization criterion. Second, linear boundaries of all the spatial objects were extracted and buffered within each data layer. Notably, the discrepancy between the data layers, acquired at various scales and data precision, was solved by adopting a generalization using two-sided buffers of 25–50 m along the borderlines within all 6 data layers. With this assumption, they were treated as overlapping when their borderlines were less than 50 m away for the source data at the scale of 1:50,000 and 100 m away for the data at lower scales, which was an inevitable approximation. Notably, with this solution, it was possible to construct the landscape contrast map from varied scale data, and at the same time, it delivered satisfying spatial accuracy. Later, all the unique pairs of buffers were multiplied, which outputs 15 variants of overlapping layers. This step was performed in GRASS GIS, which enabled efficient parallel computations of up to 4 variants simultaneously. In ArcMap, a regular grid of squares of 50 by 50 m was generated within the extent of the study area, and all the previously developed buffer overlap layers were sampled to the grid cells. Later, again within ArcMap, all these layers were merged, and the number of overlaps encountered within each cell was simultaneously recounted. The resulting data layer was the basis of the landscape contrast map constructed and visualized with QGIS, which contained cells only within areas where at least two data layers overlapped. However, their number was further limited to grid cells of the highest landscape contrast within the final composition of the landscape contrast map before the later delineation of microregional boundaries (Fig. 10).

**Fig. 11:**
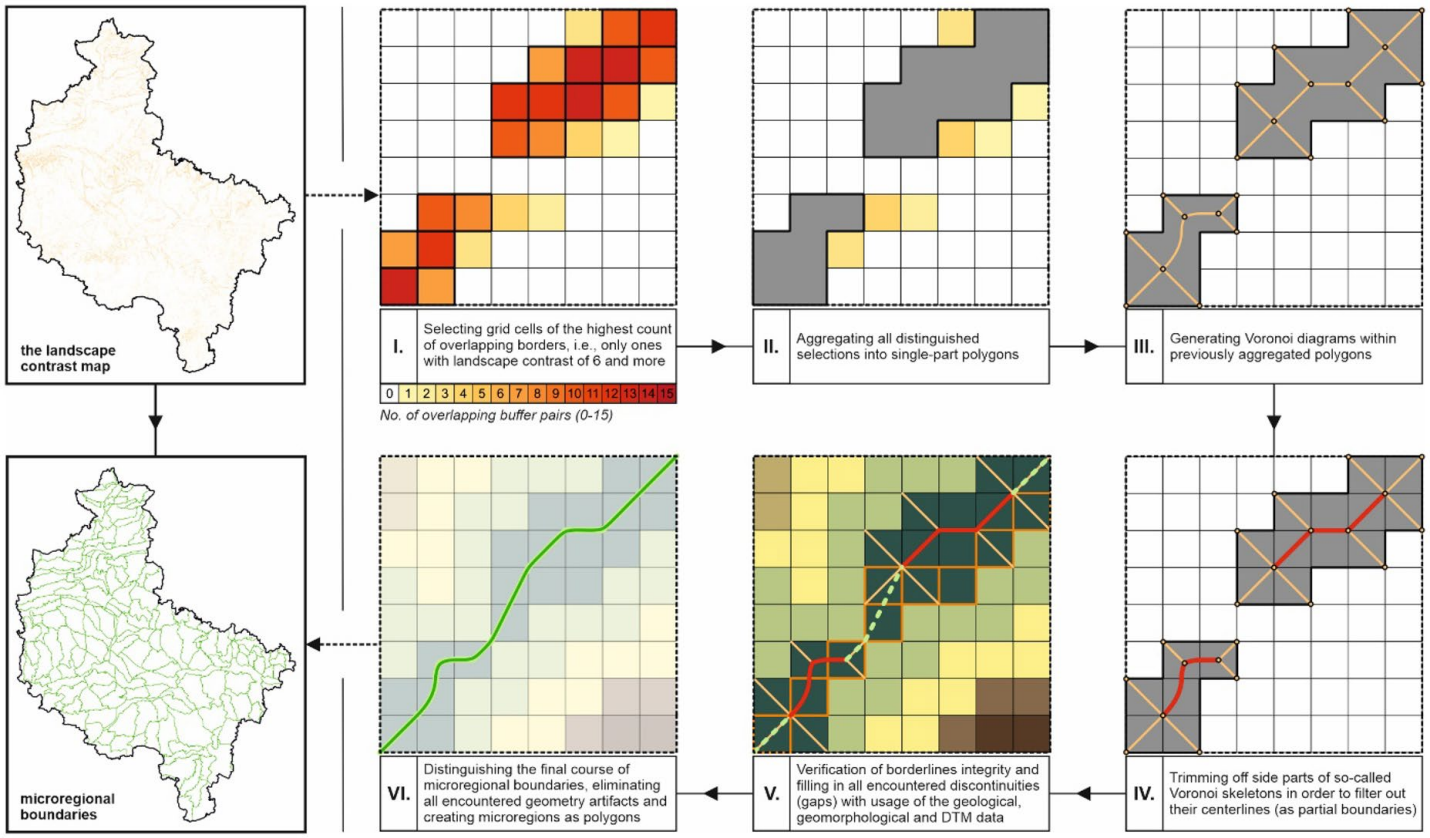
The procedure scheme of distinguishing microregional boundaries out of the landscape contrast map

The procedure of distinguishing microregions based on the developed landscape contrast map can be described in a few steps. First, only the grid cells with the highest landscape contrast were selected and aggregated into polygons. All actions were performed within the Esri ArcMap environment. When delineating boundaries, the lowest considered number of overlapping buffers was established as 6, which helped to delimit 187,082 out of 206,824 segments of the microregional boundaries directly using the landscape contrast map (more than 90%). These segments were delineated by the centerlines of the previously aggregated polygons, which were calculated using Voronoi diagrams. The remaining segments were so-called gaps (less than 10%), i.e., spatial discontinuities. These were manually filled based on the additional expert analysis of the geological and geomorphological boundaries used, including DTM data. Performing the procedure resulted in the final shape of the microregional boundaries of the Greater Poland Voivodeship (Fig. 11).

## Spatial data and processing issues

Preparation of unified spatial data layers within the territory of the Greater Poland Voivodeship required combining data of various cartographic characteristics acquired from numerous diverse sources (see Table 1). Not all thematic maps used were distributed in a unified vector format. Many were acquired as scans of previously printed maps, and their sheets required vectorization before further usage within the regionalization procedure. Varied data availability for distinct parts of the country, as well as uneven map scales or spatial data accuracy, which are frequently stored in mixed data types and file formats, is one of the general problems with the regionalization of the whole country. Moreover, to date, other parts of Poland lack some complimentary spatial data, e.g., consistent and precise genetic interpretation of landforms, which was built from scratch (Piniarski, 2020; Macias & Bródka, 2021) based on the geomorphological map of the Wielkopolska-Kujawy Lowland (Karczewski et al., 2007).

**Table 1:** Spatial data used for the Greater Poland Voivodeship microregionalization

No.	Spatial data layer	No. of spatial objects	Source data			
			*Resource*	*Map scale or corresponding data precision*	*Publication*	*Data type*
1	Subsurface geological structure	39,379	Detailed Geological Map of Poland (SMGP)	1:50,000	PGI (1956–2009)	Raster & vector
2	Genetic interpretation of landforms	5,154	Geomorphological map of the Wielkopolska-Kujawy Lowland and geomorphological drafts to Detailed Geological Map of Poland (SMGP)	1:50,000	Original, printed map by Krygowski, Ed. (1953)	Raster & vector
					Numerical map version by Karczewski et al. (2007)	
					Geomorphological drafts to SMGP (PGI, 1956–2009)	Raster
3	Terrain slope classes	56,936	Digital terrain model (DTM)	1 × 1 m grid generalized to 50 × 50 m	GUGiK (2016)	Text grid
4	Surface water and depth to groundwater	24,547	Hydrographic map of Poland	1:50,000	GUGiK (1985–2017)	Raster & vector
			Topographic Objects Database (BDOT10k) — “surface water” class	1:10,000	CODGiK (2016)	Vector
5	Genetic soil types	14,189	Soil and agricultural map	1:100,000	IUNG (2002–2010)	Vector
6	Potential natural vegetation communities	2,884	Map of potential natural vegetation	1:300,000	Matuszkiewicz (2008)	Vector

Eventually, the spatial data consisted of 6 thematic layers, i.e., one unified layer for every delimitation criterion (see Table 1). Each of them includes thousands of spatial objects, i.e., separated areas of specific environmental characteristics — homogeneous from the point of view of selected components of the environment, i.e., geological surface types (39,379), genetic landforms (5154), terrain slopes (56,936), surface waters and depth of ground waters (24,547), genetic types of soils (14,189), and potential natural vegetation (2884) (PNV). Spatial data at a resolution of 50 m within the extent of the study area include ca. 12 million test fields for each of the 6 delimitation criteria, which implies processing a total of ca. 72 million regular (square) test fields of 2500 sqm. Accordingly, constructing the landscape contrast map involved processing all 15 individual grid layers containing ca. 180 million test fields (within the multiplying buffers process). Ultimately, their number was decreased by selecting only those with boundary intersections. Within all 6 data layers, it out-turns a more than 5 times reduction of the grid cells number — to ca. 13 million test fields. By sampling all layers of the spatial data into a regular grid of squares within the spatial coverage of the study area, which simultaneously means generalizing all of them, it was possible to remove all redundant data and simplify the computations. After merging all the grids into a single grid while simultaneously recounting the number of boundaries overlapping in each grid cell, limiting their number to less than 5 million test fields was possible. Nonetheless, it was still challenging and required much computational effort from the available computing platform, especially since it first needed the initial preprocessing of all 180 million test fields to filter out all the redundant data.

## Discussion

Significantly, as a signatory of the European Landscape Convention, Poland was obliged to implement new landscape management regulations, introducing the landscape audit to the Polish law order (Karpus, 2016; Krajewski & Solecka, 2019; Szlachetko, 2019). Reviewing the literature on the subject showed the high impact of the introduced regulations on academics and their research directions in recent years. After forming initial assumptions about the landscape audit (Degórski et al., 2014; Myga-Piątek et al., 2015; Pukowiec-Kurda & Myga-Piątek, 2017; Solon et al., 2014, 2015), the scientific community of Poland’s landscape ecologists broadly debated the direction of their upcoming works devoted to the country’s physical-geographical regionalization (Kistowski et al., 2018) as a baseline for further landscape typologies and in determining priority landscapes. First, it was acclaimed that the original physical-geographical boundaries (Kondracki & Richling, 1994) needed verification and adjustment based on precision mapping using GIS solutions and up-to-date, high-resolution spatial data. Second, the necessity of developing a microregional division of the whole country area was stated, as physical-geographical microregions are perceived as the most appropriate basic test fields for local-scale landscape analysis and environmental management (Balon & Krąż, 2013; Macias et al., 2020). However, the latest revision of the physical-geographical division of Poland still does not include units in the rank of microregions (Solon et al., 2018; Richling et al., 2021).

Developing a unified, low-rank spatial natural division of the whole country appeared challenging due to numerous problems encountered within the microregionalization procedure (reviewed within the paper), most of which were not yet addressed and resolved besides the Greater Poland Voivodeship (Piniarski, 2020; Macias & Bródka, 2021). Notably, the physical-geographical microregionalization of the Kuyavian-Pomeranian Voivodeship was published before (Kot, 2015). Although GIS solutions were used, they were primarily based on traditional expert methods and simple visual analysis of the gathered spatial data. Moreover, the research did not try to provide any universal procedure that could be adopted for other parts of the country. Therefore, the Kuyavian-Pomeranian Voivodeship microregionalization could be accomplished with relatively little work and time. Its delimitation criteria include origin and morphometry, geology (lithology and its genesis), and land cover (mainly the presence of forests, agricultural land, and surface water), which correspond to basic assumptions of the physical-geographical regionalization of Poland (broadly discussed within the paper). The materials used were cartographic attachments to various studies of the local environment, i.e., thematic maps at different scales and levels of generality, mostly maps at scales from 1:100,000,000 to 1:100,000. Other resources used were DEM at a resolution of ca. 90 m and land cover data, i.e., Corine Land Cover (EEA CLC2006) at a resolution of 100 m (Kot, 2016).

Consequently, the resulting map of the Kuyavian-Pomeranian Voivodeship microregions was published only at a scale of 1:500,000 (Kot, 2015), i.e., 10 times lower than later revised mesoregions (Solon et al., 2018). Thus, their course should be verified and adjusted to correspond to the map at a scale of 1:50,000 or more (to make them useable within the landscape audit or in any other local-scale landscape analysis). According to the research, among the reviewed spatial data resources, it could be done using more detailed base maps, including subsurface geological structures (based on the SMGP) and topographic terrain attributes (acquired from DEM of the higher resolution), which are considered as guiding cartographic materials, necessary for delineating microregional boundaries (Kistowski, 2018). However, gathering and processing all the required data layers within the desired study area would still be problematic, as it was with spatial data processing within the microregionalization procedure of the Greater Poland Voivodeship (Piniarski, 2020; Macias & Bródka, 2021).

Another noteworthy attempt was the microregionalization proposal of the Silesian Voivodeship (Nita et al., 2016), which included verification of the method on selected mesoregions (Kondracki & Richling, 1994), resulting in verifying and adjusting the higher rank units within the study area to a scale of 1:50,000. Although the microregionalization of the Silesian Voivodeship still needed to be completed, the proposal results were already integrated within the revision of the physical-geographical mesoregions of Poland by Solon et al. (2018). Again, the SMGP and DTM (at resolutions of 25–75 m) were the leading spatial data resources used. Additionally, geomorphological maps were used (where available), along with other topographic, orthophoto, and thematic maps corresponding to a scale of at least 1:50,000 (as far as possible). Apart from the discussed provinces, microregional divisions of a few original mesoregions were also proposed within studies on the division of Poland into physical-geographical regions (Kistowski et al., 2018). Importantly, their findings are consistent with the author’s research, confirming the necessity of using criteria and spatial data similar to those used within the microregionalization of the Greater Poland Voivodeship, again emphasizing the need for more comprehensive implementation of GIS solutions based on traditional methodological approaches (Piniarski, 2020).

All the discussed studies implemented some GIS solutions to the traditional methodologies related to the physical-geographical regionalization of Poland. However, so far, only the microregional division of the Greater Poland Voivodeship has been accomplished within the whole province area with the precision desired for landscape auditing (Piniarski, 2020; Macias et al., 2020; Macias & Bródka, 2021). Moreover, with the research for the first time comes the proposal of a universal solution of GIS-based microregionalization of the whole country area (with a step-by-step GIS procedure and a suggestion of regionalization criteria with their corresponding spatial data). Simultaneously, each discussed study encountered problems gathering all the necessary spatial data concerning its characteristics, i.e., theme content, spatial coverage, spatial accuracy, data type, and distribution format, and further processing with the desired precision. This makes data processing and performing GIS-based procedures challenging, especially regarding vast areas and spatial data concerning many environmental characteristics.

Considering vector data, irregular geometries of high-precision spatial datasets consist of compounded topological data structures with numerous geometry nodes, making their processing more demanding and time-consuming. A topological structure of vector data is processing-intensive and static, i.e., any updating or editing of the vector data requires rebuilding an entire topology. For this reason, vector data algorithms are often complex and processing-intensive, which innately limits their functionality for processing large datasets (Buckley et al., 1990). In general, raster data are stored using a compact data structure, and it is easier and more computationally efficient to manipulate them with GIS software (Silva-Coira et al., 2020). Therefore, another analytical problem within the regionalization procedure is processing large, vector format spatial datasets, which needs to be improved (Piniarski, 2020). The most time-consuming stage within the microregionalization procedure was identifying common areas of the overlapping buffers, where each of the 15 multiplying operations took 38 to 56 h of uninterrupted computations. GRASS GIS made it possible to compute up to 4 variants simultaneously on a single computing unit. Even though every single operation was limited to only one CPU thread, the load was always only up to 50–60% of its potential computing power, and only one-third of the accessible RAM was permanently in use. Therefore, better-optimized GIS tools, including native implementation of parallel computations and support for hardware acceleration, e.g., general-purpose computing on graphics processing units (GPGPU) technology (Zhang & You, 2012; Stojanovic & Stojanovic, 2013, 2014; Prasad et al., 2015; Zhou et al., 2017; Breunig et al., 2020; Saupi Teri et al., 2022), would be beneficial in future studies related to regionalization of the whole country. Nevertheless, findings resulting from the microregionalization of the Greater Poland Voivodeship (Piniarski, 2020) could be a reasonable baseline for preparing all the necessary spatial datasets and successfully implementing the developed GIS procedure for creating a microregional division of all the Polish provinces and, finally, the whole country area.

## Conclusions

Foremost, a GIS-based physical-geographical microregionalization standard has yet to be set in Poland. Thus, the developed regionalization procedure still has the potential to become a universal solution, i.e., set a new standard for microregionalization for all Polish provinces and finally shape the course of microregional boundaries all over the country. Importantly, the Greater Poland Voivodeship microregionalization was the first fully accomplished elaboration, which was complementary to the works connected with the landscape audit and, among others, was used to determine priority landscapes within the study area. However, developing a microregional division of the Greater Poland Voivodeship has revealed some hard-to-overcome difficulties, which would undoubtedly occur in the microregionalization of the rest of the country, mainly concerning spatial data availability and processing.

First, many available spatial data resources vary in quality within different parts of the country. Second, their limited spatial coverage and fragmentation imply the necessity of combining numerous resources, i.e., preprocessing and unifying all the source data before their final analysis. Third, so far, there are no spatial data that would correspond to all selected delimitation criteria in the desired spatial accuracy and would already be unified for the entire territory of Poland. To date, all the required spatial data have been developed for approximately 10% of the country area, especially for the microregionalization of the Greater Poland Voivodeship. Therefore, complementary spatial data must be acquired before the microregionalization of the remaining provinces.

Additionally, one of the general problems with physical-geographical regionalization is the subjective choice of methods, delimitation criteria, and corresponding spatial data. Regionalization results and their further interpretation critically depend on these choices. Therefore, the regionalization procedure must implement GIS solutions within the existing, commonly accepted fundamentals. The theoretical background and guidelines of Polish physical-geographical regionalization have a long tradition and have become universally approved by the government, researchers, and practitioners nationwide.

**Fig. 12:**
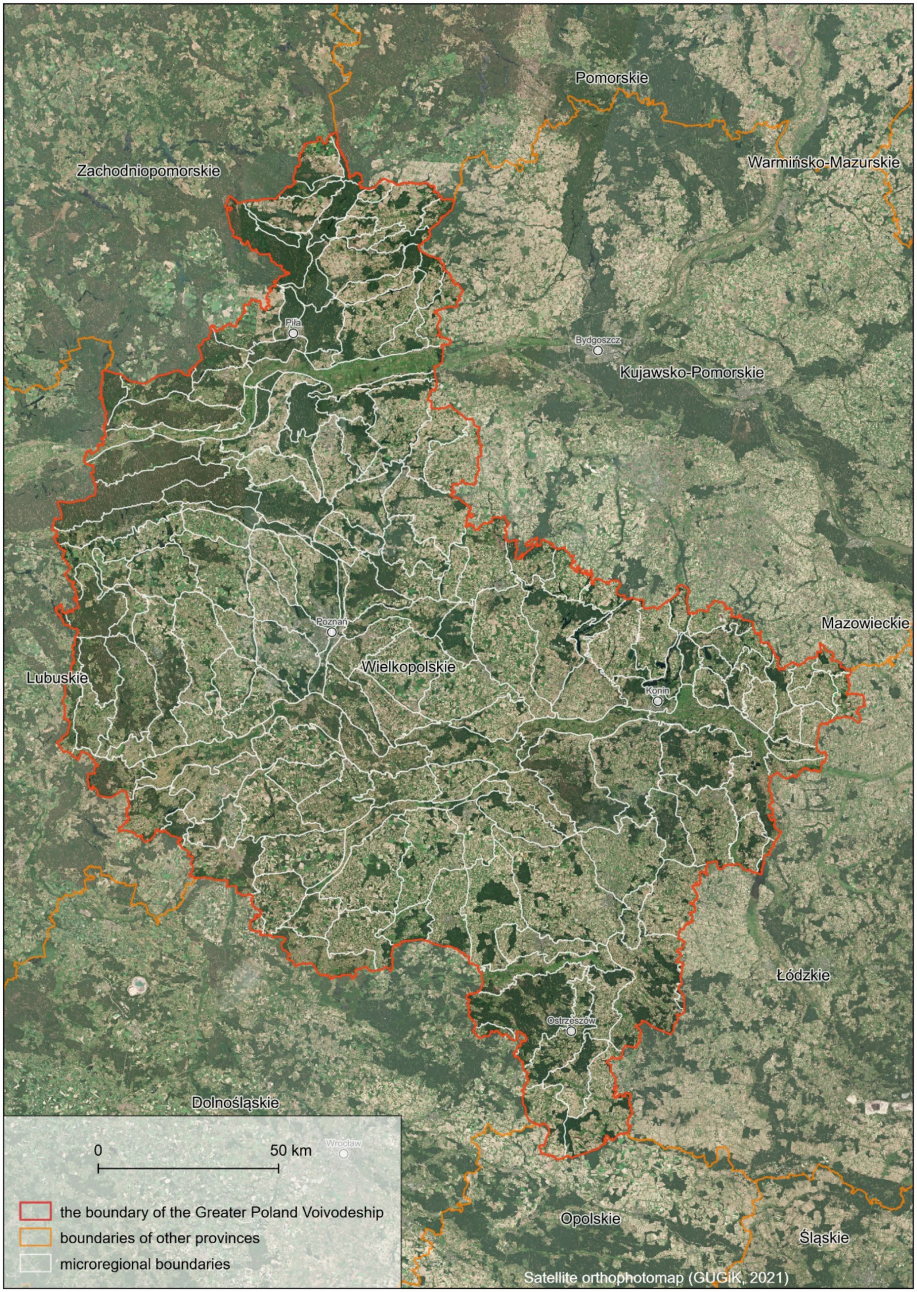
The final shape of microregional boundaries distinguished within the Greater Poland Voivodeship

Although GIS-based physical-geographical microregionalization of the Greater Poland Voivodeship occurred computationally demanding, the landscape contrast analysis method has proven to be a workable solution for delineating low-rank natural spatial units (Fig. 12). Notably, the procedure efficiency could essentially benefit from better optimization of GIS tools, which still require improvements, e.g., broader implementation of parallel computing and support for hardware acceleration of data processing.

The microregionalization process should proceed for the rest of the provinces to complete the GIS version of the physical-geographical regionalization of Poland by Solon et al. (2018). The local government already used the microregionalization of the Greater Poland Voivodeship in their works related to landscape auditing (Piniarski, 2020; Macias et al., 2020; Macias & Bródka, 2021), which resulted in the landscape audit resolution of the Greater Poland Voivodeship, which was successfully adopted by the Greater Poland Voivodeship Sejmik (regional assembly) in 2023 (Resolution No. LI/1000/23 SWW of 27 March 2023).

Moreover, some early works related to the microregionalization of the Lubusz Voivodeship (Lubuskie Province) using the developed scheme are already in progress. Notably, the developed concept was accepted by the local governments and appreciated by the landscape ecologist’s community, awarded third place in the IALE 2022 European Landscape Ecology Congress poster competition on 11–15 July 2022 in Warsaw (Poland).

## Data Availability

All the datasets generated during the current study are available from the corresponding author upon reasonable request.
